# Effectiveness of short active breaks for reducing sedentary behavior and increasing physical activity among Japanese office workers: one-year quasi-experimental study

**DOI:** 10.5271/sjweh.4224

**Published:** 2025-07-01

**Authors:** Naruki Kitano, Takashi Jindo, Kaori Yoshiba, Daisuke Yamaguchi, Yuya Fujii, Kyohsuke Wakaba, Kazushi Maruo, Yuko Kai, Takashi Arao

**Affiliations:** 1 Physical Fitness Research Institute, Meiji Yasuda Life Foundation of Health and Welfare, Tokyo, Japan.; 2 Division of Art, Music, and Physical Education, Osaka Kyoiku University, Kashiwara, Osaka, Japan.; 3 Faculty of Human Life, Jumonji University, Niiza, Saitama, Japan.; 4 Department of Biostatistics, Institute of Medicine, University of Tsukuba, Tuskuba, Ibaraki, Japan.

**Keywords:** accelerometer, occupational health, presenteeism, propensity score, psychological distress, work engagement

## Abstract

**Objectives:**

We examined the effects of a one-year multicomponent workplace intervention that introduced short active breaks from prolonged sitting on occupational movement behaviors and health among Japanese office workers.

**Methods:**

This quasi-experimental study was conducted in Tokyo, Japan (2019–2020). In the intervention group (N=172), activity breaks from sitting were introduced to the work schedule (approximately 10 minutes/working hour) together with support strategies to encourage participation (eg, social support, provision of information). Workers in the control group (N=323), who worked at the same company group as those in the intervention group, did not receive any intervention. We evaluated accelerometer-measured sedentary behavior and physical activity during working hours as primary outcomes, and mental health and subjective job performance as secondary outcomes. Propensity score weighting using overlap weights was performed to examine between-group differences in outcomes at one year.

**Results:**

At the one-year follow-up assessment, sedentary behaviors during working hours in the intervention group decreased by 24.4 minutes (95% confidence interval 31.6–17.3), with physical activity increasing by a comparable amount (P for group difference <0.05). However, at the one-year follow-up, psychological distress had worsened and work engagement had declined in the intervention group relative to baseline (P for group difference <0.05).

**Conclusions:**

Our findings suggest that this program is a feasible approach to reducing sedentary behavior and promoting physical activity during work hours among office workers. However, methodological limitations prevent the definitive attribution of the effects to the intervention. Further rigorous research is needed to assess its effectiveness and external validity before broad implementation.

Office workers, who represent a substantial proportion of the modern workforce, spend more time engaging in sedentary behavior (SB) than workers in other occupations (1). Most of their daily SB occurs in the workplace (2), comprising long bouts of uninterrupted sitting (3). As excessive sitting causes various adverse health events (eg, chronic diseases, mental illness, premature death) (4–6), researchers have studied strategies to reduce it and increase physical activity (PA) during work hours among office workers.

A systematic review revealed greater reductions in occupational sitting with interventions involving physical workplace changes (eg, introduction of sit-stand desks, active workstations) or a combination of physical changes and educational and behavioral interventions (7). However, the effectiveness of these environmental interventions in reducing SB may diminish three months after implementation (8). Their cost-effectiveness is also undetermined and may discourage employers from implementing them (9). To address the social challenges of excessive sitting among office workers, the development of workplace health promotion programs with lower implementation costs is important, even if their effectiveness is not maximized.

One alternative is to change workplace policies to incorporate short active breaks from sitting within the organizational schedule. However, the effects of introducing short active breaks on occupational SB have only been examined in two randomized controlled trials (RCT) (10, 11). Moreover, the results were inconsistent, with one revealing reduced SB on workdays (10) and the other finding no strong evidence for reducing total or occupational SB (11). Consequently, a systematic review could not determine the effectiveness of such a workplace policy change aimed at reducing SB, and further research was recommended (8). Three further limitations exist in this research topic. First, previous intervention studies lasted ≤6 months, and real-world longer-term effects remain unclear. Second, social norms and organizational culture (12, 13), which are determinants of workplace strategies to reduce SB, vary across countries/regions (14). Thus, research across multiple regions is needed to establish this type of intervention’s external validity and effectiveness. Third, systematic reviews have revealed that workplace health promotion, including PA promotion, positively affects mental health and work engagement (15, 16). Short active breaks, particularly when taken with coworkers, could improve mood, strengthen workplace relationships, and foster group cohesion, ultimately leading to reduced psychological distress and increased work engagement. However, to the best of our knowledge, the effects of introducing such breaks on these outcomes—which are essential in today’s working population—remain unexplored (17, 18).

Therefore, we examined the effects of a one-year multicomponent intervention introducing short active breaks within the work schedule on occupational SB and PA among Japanese office workers and assessed its potential impact on mental health and work-related outcomes.

## Methods

This study followed the Transparent Reporting of Evaluations with Non-randomized Designs (TREND) statement (supplementary material, www.sjweh.fi/article/4224, table S1).

### Participants and study design

This one-year quasi-experimental study was conducted between 2019 and 2020. The inclusion criteria for the intervention and control groups were: (i) being an office worker, (ii) not using a sit-stand desk, and (iii) working during 09:00–17:00 hours per the company’s work regulations (ie, no flex-time work). Workers in the intervention group worked for a company that provided insurance administration services with offices in urban areas such as Tokyo and Osaka, Japan. The intervention was implemented in all departments and for all the company’s workers (23 offices; 1009 office workers). However, to reduce the burden on the company and its employees, the trial was conducted among a subset of 200 employees from two departments, arbitrarily selected by accompanying personnel. Of these, 190 workers provided consent for, and completed, the baseline survey. Finally, 172 workers participated in the survey one year after the intervention began and their data were analyzed.

**Figure 1:**
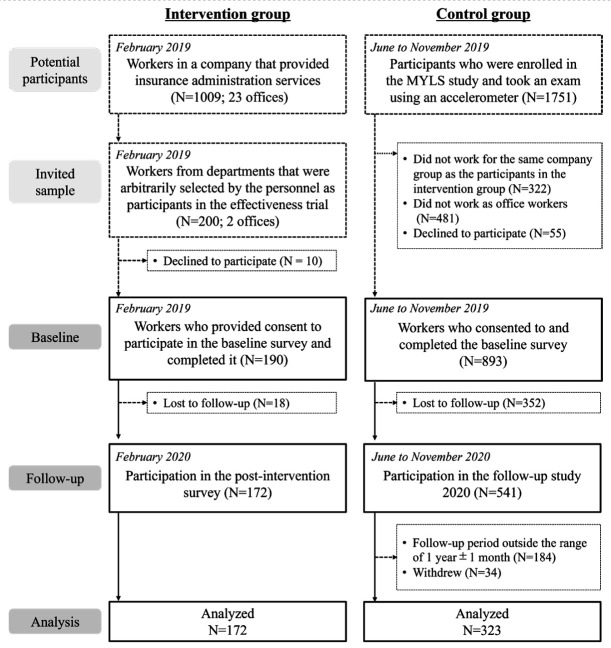
Flow diagram of participant selection. [MYLS=Meiji Yasuda LifeStyle.]

For the control group, we assigned participants from a cohort study, the Meiji Yasuda LifeStyle (MYLS) study (19). The MYLS study used data from the annual health checkups of employees living in the Tokyo metropolitan and surrounding areas (ie, same area as the intervention group participants’ office). To enhance comparability with the intervention group, we included participants who met the following criteria: (i) worked for the same company group as the participants in the intervention group, (ii) worked as office workers, (iii) had data in 2019 (ie, when the intervention began) and 2020 (ie, when the intervention ended), and (iv) had a follow-up period of 365 [standard deviation (SD) 30] days (approximating the intervention period). Ultimately, 323 workers met these criteria and were included (figure 1).

The follow-up survey was conducted at the time of the coronavirus disease (COVID-19) pandemic (intervention group: February 2020; control group: June to November 2020). In Japan, strict policies such as lockdowns were not implemented. Instead, social distancing measures were adopted. During the post-measurement period for both groups, no strict measures were implemented that would have significantly affected people’s movement behaviors (supplementary figure S1). In the control group companies, work-from-home policies were adopted; however, owing to insufficient infrastructure, >80% of employees worked from the office. No restrictions were placed on coming to work as a countermeasure against infection.

### Intervention

The one-year multicomponent intervention program that introduced short active breaks was developed based on the Social Ecological Model (20) and the COM-B model (21). Supplementary figure S2 shows the intervention timeline. Workers in the control group did not receive any intervention from the research team and continued to work as usual for one year.

### Supporting strategies

Previous studies have revealed the keys to success in workplace health promotion: understanding among management, a need among employees, and early involvement of management and employees in the program’s implementation (12, 13, 22, 23). Hence, before implementing the short breaks, the company’s management worked with the researchers to build a conducive climate (February 2018 to January 2019). These efforts included a survey to explore the employees’ health issues and the provision of feedback on its results, encouragement of short active breaks by the CEO of the company, the provision of information and education to interrupt prolonged periods of sitting at the office, and allocating leaders in each department to promote and develop the program. Supplementary material S1 details the educational material distributed to the each department and to the employees.

### Introducing short active breaks from sitting

The activity breaks were aimed at reducing sitting by approximately ten minutes daily. The leaders of each department developed a break program based on their work schedules and needs. Regarding the program, participants had the freedom to undertake activities of their choice during their break times but were required to stand up/walk around, and the goal was to reduce SB and increase PA for a total of ten minutes per workday. Participants were not forced to take active breaks. For example, one department developed a program comprising five minutes of stretching/walking at any time from 10:00–11:00 hours (depending on individual/group availability) and five minutes of light-intensity aerobic exercises at 14:00 hours, for a total of ten minutes per workday (supplemental table S2). Each department’s developed break program involved all the relevant employees and was implemented under the name “Kenkatsu★Time” from February 2019 onward. During the intervention period, the management and researchers recommended periodic revision of the program by each department to ensure its long-term sustainability. For example, in one department, the types of activities (eg, stretching, light gymnastics), starting signal, and teams in which breaks were taken slightly changed one year after the intervention began. However, at the time of the follow-up survey, the goal of reducing SB by at least ten minutes/day had been maintained.

### Measurements

For the intervention group, data were collected at two time points: one week before the intervention began (January 2019) and one year after the intervention began (February 2020). For the control group, data from June to November 2019 and from June to November 2020 were collected.

### Primary outcomes

The primary outcomes were changes in occupational SB and PA assessed using a validated triaxial accelerometer (Active style Pro HJA750-C, Omron Healthcare, Kyoto, Japan) (24–26). The participants in the intervention and control groups were instructed to wear the device on their waist from the beginning to the end of the workday and during the waking hours, respectively, for ≥10 days. For the control group, activity data during working hours (ie, 09:00–17:00 hours) were extracted as follows. Non-wear time was defined as an interval of 60 consecutive minutes with activity counts below the detection limit. Valid days were defined as a day when participants wore the device for ≥75% of the working hours (27). The data of workers with ≥4 valid days were used in the analysis (28), and data for those who did not meet this criterion were imputed using the procedure described below. Each 60-second epoch was classified as SB [≤1.5 metabolic equivalents (MET)], light-intensity PA (LPA; 1.6–2.9 MET), or moderate-to-vigorous–intensity PA (MVPA; ≥3.0 MET) (29, 30). We also calculated indicators related to the short active breaks: the frequency of and time spent in prolonged SB (time accumulated in prolonged, unbroken sitting bouts ≥30 minutes) and bouted PA [defined as continued PA ranging from 3 minutes (the typical single break session) to 10 minutes (the daily goal)]. At each survey point, these indicators were aggregated per day, standardized to an 8-hour workday [*standardized minutes=observed minutes in each behavior/wearing minutes*×*480]*, and averaged over all the valid days.

### Secondary outcomes

The secondary outcomes were psychological distress, work engagement, and subjective job performance, which were assessed using a self-report questionnaire. The K6 scale (31) was used to evaluate psychological distress (0–24 points; higher scores indicate worse psychological distress). The Utrecht Work Engagement Scale (32) was used to assess work engagement, a positive psychological state regarding work (0–6 points; higher scores indicate better engagement). To assess subjective job performance, we used a question on absolute presenteeism from the World Health Organization Health and Work Performance Questionnaire (33) (0–10 points; higher scores indicate better performance). Supplementary table S3 provides detailed information on each indicator.

### Adherence to the short-break program

Adherence to short active breaks was assessed during the follow-up survey. Participants were asked to recall their adherence at the following three time points: (i) when the short breaks were first implemented in each department, (ii) six months after implementation, and (iii) one year after implementation. The response options were "almost never", "sometimes", "mostly", and "almost always".

### Other variables

Although workers in the control group were selected from the same company group as those in the intervention group, the participants’ characteristics might have differed across the groups. Therefore, to enhance the comparability of the two groups, the following data were investigated and used in the statistical analysis as variables to balance characteristics across the groups: age, sex, body mass index (BMI), and job position.

### Statistical analysis

In non-RCT, statistical methods such as propensity score (PS) matching and inverse probability weighting are considered useful tools for causal inference. However, these methods have limitations concerning extreme PS values and extreme weighting (eg, off-support observations and extremely small/large weights). Hence, to examine the effect of our multicomponent program on the outcomes, PS weighting using overlap weights (control group: PS; intervention group: 1-PS) was applied to examine the average treatment effect for the overlapping population (ATO) (34). The calculated weights are smaller for extreme PS values to ensure that outliers (ie, those who are nearly always treated/never treated) do not dominate results and worsen precision. Therefore, these outliers contribute relatively little to the estimates, while participants whose characteristics align with either group contribute relatively more. The resulting target population mimics the characteristics of a pragmatic randomized trial that is highly inclusive, not excluding any study participants from the available sample but emphasizing the comparison of patients at clinical equipoise (35). The *PSweight* package was used for analysis (36). For the inference of ATO, the dependent variable in the model was the change in each outcome from baseline to the follow-up survey, and the sandwich variance estimator was used. Logistic regression analysis was used to calculate the PS, with the group as the dependent variable and age, sex, BMI, job position, and baseline values for each outcome as the independent variables.

We performed multiple imputation by chained equations using predictive mean matching to impute all missing data using the *mice* package (37). Twenty imputed datasets were generated and analyzed individually. The results from each dataset were pooled according to Rubin’s rule. As a sensitivity analysis, the aforementioned model was reanalyzed using the complete case. All statistical analyses were performed using R version 4.2.2 (R Foundation for Statistical Computing, Vienna, Austria). Statistical significance was set at P<0.05.

## Results

### Participant characteristics

The analytical sample comprised data from 172 and 323 workers in the intervention and control group, respectively. Except for the time spent doing MVPA and the step count during working hours, significant between-group differences were found in the baseline demographics and outcome variables (table 1). The mean (SD) age and occupational SB time in the intervention and control group were 45.3 (11.6) and 51.6 (7.2) years, and 308.1 (62.7) and 341.4 (56.7) minutes, respectively. Supplementary figure S3 shows the PS distribution in each group. After PS weighting using overlap weights, these variables were balanced across the groups (supplementary figure S4).

**Table 1:** Characteristics of the study participants. [SB=sedentary behavior; SD=standard deviation; LPA=light-intensity physical activity; MVPA=moderate-to-vigorous-intensity physical activity.]

Characteristic		Intervention (N=172)			Control (N=323)		P-value ^a^
		Mean (SD)	N (%)		Mean (SD)	N (%)	
Age (years)		45.3 (11.6)	–		51.6 (7.2)	–	<0.01
	Missing	6	–		0	–	
Women		–	157 (92.4)		–	182 (56.3)	<0.01
	Missing	–	2		–	0	
Body mass index (kg/m^2^)		21.9 (3.3)	–		23.3 (4.4)	–	<0.01
	Missing	17	–		0	–	
Manager		–	24 (14.1)		–	91 (28.2)	<0.01
	Missing	–	2		–	0	
**Accelerometer-obtained variables**							
Wearing days (days)		4.7 (0.5)	–		12.2 (4.5)	–	<0.01
	Missing	28	–		17	–	
Weartime (min/8 hrs)		442.8 (30.8)	–		478.9 (6.1)	–	<0.01
	Missing	28	–		17	–	
SB (min/8 hrs)		308.1 (62.7)	–		341.4 (56.7)	–	<0.01
	Missing	28	–		17	–	
Prolonged SB (min/8 hrs) ^b^		64.2 (67.7)	–		115.3 (83.0)	–	<0.01
	Missing	28	–		17	–	
Prolonged SB (counts/8 hrs) ^b^		1.5 (1.4)	–		2.4 (1.5)	–	<0.01
	Missing	28	–		17	–	
LPA (min/8 hrs)		149.8 (56.7)	–		116.0 (53.0)	–	<0.01
	Missing	28	–		17	–	
MVPA (min/8 hrs)		23.1 (13.1)	–		23.7 (13.5)	–	0.691
	Missing	28	–		17	–	
Bouted PA (min/8 hrs) ^c^		75.8 (34.2)	–		57.6 (33.1)	–	<0.01
	Missing	28	–		17	–	
Bouted PA (counts/8 hrs) ^c^		17.1 (7.0)	–		13.2 (6.9)	–	<0.01
	Missing	28	–		17	–	
Step count (steps/8 hrs)		3834.5 (1313.6)	–		3597.2 (1385.8)	–	0.080
	Missing	28	–		17		
Psychological distress (points)		5.1 (5.2)	–		3.1 (4.0)	–	<0.01
	Missing	3	–		2	–	
Work engagement (points)		2.2 (0.9)	–		2.9 (1.5)	–	<0.01
	Missing	3	–		8	–	
Job performance (points)		5.0 (1.3)	–		6.1 (1.6)	–	<0.01
	Missing	4	–		1	–	

^a^ We performed Welch’s two-sample t-test for continuous variables and Pearson’s chi-square test for binary variables. ^b^ Unbroken sitting bouts ≥30 minutes. ^c^ Continued physical activity of 3–10 minutes.

### Short-break program adherence

**Figure 2:**
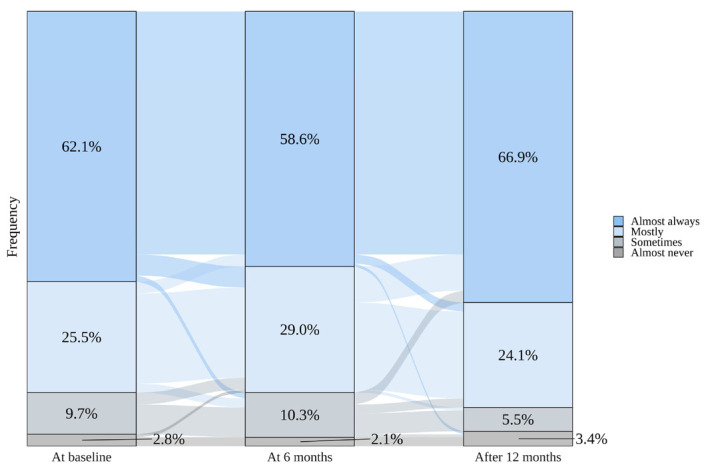
Adherence to the program incorporating short active breaks from sitting in the intervention group. The graph was created using data from 145 participants without any missing data at the three time points.

Figure 2 shows the adherence to the short active breaks over a year. More than half of the workers *almost always* participated in the break, with an increase in the later stages of the intervention: 62.1% immediately after program implementation, 58.6% after 6 months, and 66.9% after 12 months.

### Intervention effects

Table 2 shows the effects of the intervention on SB and PA during working hours. At the one-year follow-up, workers in the intervention group decreased SB and increased PA during working hours (P for group difference <0.05). The intervention group saw a 24.4-minute decrease in SB time [95% confidence interval (CI) 31.6–17.3], a 12.6-minute increase in LPA (95% CI 6.3–18.9), and a 10.1-minute increase in MVPA (95% CI 8.0–12.2). The frequency of and time spent in prolonged SB decreased, although not significantly (P=0.050–0.059), and short-bouted PA increased (P<0.001). The control group showed no significant changes in either primary outcome.

**Table 2:** Effect of the multicomponent intervention incorporating short active breaks from prolonged sitting on the primary outcomes. [SB=sedentary behavior; LPA=light-intensity physical activity; MVPA=moderate-to-vigorous-intensity physical activity; SD=standard deviation; CI=confidence interval].

Outcome	Group	Pre		Post		Post – Pre difference		Group difference	P for group difference
		Mean (SD)		Mean (SD)		Estimate (95% CI) ^a^		Estimate (95% CI)	
SB (min/8 hrs)	Intervention	309.2 (64.5)		291.1 (62.5)		-24.4 (-31.6– -17.3)		-25.9 (-34.2–-17.5)	<0.001
	Control	341.5 (56.9)		342.9 (53.2)		1.4 (-3.0–5.9)			
Prolonged SB ^b^ (min/8 hrs)	Intervention	66.5 (68.5)		65 (58.8)		-4.8 (-13.8–4.2)		-10.3 (-21.1–0.4)	0.059
	Control	114.8 (82.8)		119.9 (81.5)		5.6 (-0.3–11.4)			
Prolonged SB ^b^ (counts/8 hrs)	Intervention	1.6 (1.5)		1.5 (1.2)		-0.1 (-0.3–0.0)		-0.21 (-0.4–0.0)	0.050
	Control	2.4 (1.5)		2.5 (1.6)		0.1 (-0.0–0.2)			
LPA (min/8 hrs)	Intervention	148.2 (56.9)		155.7 (54.7)		12.6 (6.3–18.9)		14.7 (7.2–22.3)	<0.001
	Control	115.9 (52.9)		114 (50.7)		-2.1 (-6.2–2.0)			
MVPA (min/8 hrs)	Intervention	23.7 (13.7)		33.4 (16.1)		10.1 (8.0–12.2)		9.7 (7.2–12.1)	<0.001
	Control	23.5 (13.4)		23.5 (12.7)		0.5 (-0.9–1.8)			
Bouted PA ^c^ (min/8 hrs)	Intervention	72.88 (34.5)		80.3 (32.9)		10.2 (6.0–14.3)		12.1 (7.2–17.1)	<0.001
	Control	58.0 (33.2)		57.1 (31.0)		-2.0 (-4.7–0.7)			
Bouted PA ^c^ (counts/8 hrs)	Intervention	16.5 (7.1)		17.9 (6.5)		1.9 (1.0–2.8)		2.5 (1.4–3.5)	<0.001
	Control	13.3 (6.9)		13.0 (6.4)		-0.6 (-1.1–0.0)			
Step count (steps/8 hrs)	Intervention	3902.6 (1403.7)		4933.2 (1710.3)		1179.8 (948.7–1410.9)		1171.5 (884.0–1459.0)	<0.001
	Control	3589.1 (1372.8)		3630.9 (1373.2)		8.3 (-163.4–180.0)			

^a^ As these values are adjusted for covariates, they do not exactly match the difference between the values in the Pre and Post columns, which are arithmetic means. ^b^ Unbroken sitting bouts ≥30 minutes. ^c^ Continued physical activity of 3–10 minutes.

Table 3 shows the effects of the intervention on the secondary outcomes. At the one-year follow-up assessment, psychological distress [1.6 points (95% CI 0.9–2.2)] and work engagement [-0.1 points (95% CI -0.2– -0.01)] in the intervention group were significantly worsened from baseline (P for group difference <0.05). Little evidence of an intervention effect on subjective job performance was found.

**Table 3:** Effect of the multicomponent intervention incorporating short active breaks from prolonged sitting on the secondary outcomes. [SD=standard deviation; CI=confidence interval].

Outcome ^a^	Group	Pre		Post		Post - Pre difference		Group difference	P for group difference
		Mean (SD)		Mean (SD)		Estimate (95% CI) ^b^		Estimate (95% CI)	
Psychological distress (points)	Intervention	5.1 (5.2)		6.7 (5.6)		1.6 (0.9–2.2)		1.4 (0.6–2.3)	0.001
	Control	3.1 (4.0)		3.5 (4.2)		0.1 (-0.4–0.7)			
Work engagement (points)	Intervention	2.2 (0.9)		2.1 (1.0)		-0.1 (-0.2–-0.0)		-0.2 (-0.4–-0.1)	0.012
	Control	2.8 (1.5)		2.9 (1.5)		0.1 (-0.0–0.2)			
Job performance (points)	Intervention	5.0 (1.3)		5.3 (1.5)		0.2 (-0.1–0.5)		-0.2 (-0.6–0.2)	0.276
	Control	6.0 (1.6)		6.1 (1.5)		0.4 (0.2–0.6)			

^a^ Larger values indicate higher levels of psychological distress, work engagement, and job performance. ^b^ Because these values are adjusted for covariates, they do not exactly match the difference between the values in the Pre and Post columns, which are arithmetic means.

The complete case analyses (supplementary tables S4 and S5) yielded similar results, although some differences in the magnitude and variance of the estimates were found.

## Discussion

We examined the effects of a one-year multicomponent intervention that incorporated short active breaks into work schedules on occupational movement behaviors, mental health, and work-related outcomes among Japanese office workers. More than half the workers almost always participated in the break sessions throughout the year. At the one-year follow-up assessment, the time engaged in SB during working hours among participants in the intervention group decreased by approximately 20 minutes, with the time undertaking PA increasing by a comparable amount. Additionally, psychological distress and work engagement at the one-year follow-up assessment worsened from baseline in the intervention group.

The high adherence throughout the year suggests that the short-break program is feasible and sustainable in a real-world setting. A previous 8-week study involved assessing the effectiveness of stand/move breaks for 1–2 minutes every 30 minutes during working hours; 55.9% of workers participated at the end of the study (38). In another study, a multicomponent short-break program was implemented in the office (≥4 minutes/session, 4 times/day); the participants attended 31.5% of the sessions throughout the 6-month program (11). Herein, adherence (66.9% of the participants participated in nearly every break session after 12 months) was comparable to or better than that in previous studies, suggesting that our multicomponent program can persist in real-world offices over the long term (≥1 year). The higher adherence in this study than in previous studies could be explained by the supportive strategies implemented before the short-break introduction. These strategies, including social support from managers/coworkers and information provision, may have mitigated the barriers to interrupting long periods of sitting in the workplace (eg, an organizational culture unsupportive of taking active breaks) (12, 13).

Our multicomponent intervention decreased SB and increased PA during working hours by 24.3 minutes/8 hours. Only two studies have examined the effects of short-break implementation, with inconsistent results; Mailey et al (38) reported a decrease in occupational SB, whereas Akksilp et al (11) reported null effects. Our results support the findings of Mailey et al (38), who reported that 8 weeks of incorporating short active breaks reduced the time spent in SB during working hours by 35.5 minutes/9 hours (equivalent to 31.61 minutes/8 hours). These similarities may stem from the baseline occupational SB time in our participants aligning more with those reported by Mailey and colleagues than Akksilp et al. Meanwhile, the smaller changes in SB found in our study, compared to those in the study by Mailey et al, could be explained by the study period (12 months versus 8 weeks) and the components included in the intervention. This hypothesis is partially supported by the findings of a previous systematic review that suggested that the decrease in SB becomes smaller as the study period becomes longer (8).

We confirmed that our short-break program increased both LPA and MVPA during working hours, suggesting that the intervention increased the activity around the participants’ own workspaces (ie, LPA) and that of walking in and out of the office (ie, MVPA). Additionally, the observed change in SB (approximately 20 minutes/workday) was greater than the target value, which was a total reduction of approximately 10 minutes/day of occupational SB time. Possible reasons for these unexpected results include the employees’ improved literacy toward reducing SB and increasing PA in the office, which may have fostered an active organizational culture, and them attempting to complete other errands while taking active breaks (eg, hydrating, visiting the restroom, visiting a colleague) (13). These hypotheses are partially supported by our findings that short-bouted PA increased.

Our results have several implications. A previous meta-analysis has revealed that occupational SB time is reduced by 72.8 minutes/8 hours after implementing environmental workplace interventions (eg, installing sit-stand desks), by 15.5 minutes/8 hours after implementing educational/behavioral interventions (eg, counseling, providing information, computer prompts), and by 88.8 minutes/8 hours after implementing a combination of these interventions (7). Although direct comparison is difficult owing to different study methodologies, the effect of a multicomponent intervention including short active breaks on occupational SB was suggested to be comparable to educational/behavioral interventions but not as effective as environmental interventions. Meanwhile, a systematic review highlighted that many workplace interventions designed to reduce SB and increase PA are short-term in nature, ranging from a few weeks to a few months (8). Our study expands the literature by showing that our multicomponent program incorporating short breaks may be effective in reducing occupational SB and increasing PA over the long term (at least 1 year). Although further rigorous studies are needed, our intervention program—that does not entail introducing sit-stand desks or active workstations—should be considered in the population approach to reduce SB and increase PA, especially for small and medium enterprises with limited resources for employee health promotion (39).

We found no evidence that the intervention promoted health; additionally, psychological distress and work engagement in the intervention group were worse after one year, which may be intervention-related. Although the PA was brief and light, it may have caused physical or mental strain, especially among fatigued or unmotivated employees. Taking breaks may also have disrupted focused work. However, no reviews of workplace interventions, including those promoting PA, have revealed negative effects on mental health outcomes (15). Psychological distress and work engagement are affected by various factors, including job demands (eg, overtime work) and control, effort–reward imbalances, social support, and organizational change (40, 41). At the time of our follow-up survey, only the company in the intervention group was in its busy season, with increased work demands on employees, possibly resulting in worse outcomes. We assigned employees of the same company group to each group and attempted to balance the characteristics using PS weighting. However, the distribution of these outcome determinants may not have been sufficiently balanced. Future RCT should verify the observed potential negative effects.

The influence of measurements having been taken at different time points in each group on the observations and estimates must be acknowledged. Particularly, post-measurements may have been affected by the COVID-19 pandemic. For example, Japan’s primary infection control measure, social distancing, may have reduced workplace interactions and breaks, leading to lower PA and increased SB. Conversely, stair use to avoid enclosed spaces (eg, elevators) and adjustments to office traffic flow for infection control may have promoted PA. While we cannot determine the magnitude and direction of these potential effects, we also cannot exclude the possibility that observed values were influenced by differences in measurement timing. However, this influence did not seem significant enough to overturn the results, especially for the main outcome—SB and PA at work— which could be less sensitive to a pandemic than SB and PA in leisure-time would be. Moreover, in Japan, no strict measures were implemented at the time of the post-survey that would have significantly affected people’s movement behavior. Additionally, the pandemic and its countermeasures (eg, telecommuting) can reportedly worsen people’s PA (42, 43) and mental health (44). However, except for the secondary outcomes in the intervention group, no such a worsening tendency was confirmed. Meanwhile, worsened psychological distress and work engagement in the intervention group may have been influenced by anxiety regarding the onset of an unprecedented pandemic. Our findings should be validated in further RCT conducted after the pandemic.

### Strengths and limitations

A strength of this study is that we examined the long-term effects of a multicomponent intervention that incorporated short active breaks from prolonged sitting, which is expected to be effective in reducing occupational SB without having to implement environmental interventions. Additionally, we used an accelerometer to measure SB and PA and balanced group characteristics using PS weighting. However, this study also has certain limitations. The first concerns its internal validity. Although we attempted to reduce confounding bias using PS weighting, owing to the limited available data, only a small set of covariates could be included in the PS model. Therefore, our results may have been influenced by unmeasured and unknown confounders (eg, workplace culture, interpersonal relationships, awareness of health promotion). The difference in survey periods between the groups and its related factors may have also affected our results. The second limitation concerns measurement errors. The accelerometer we used has been validated for measuring PA and SB (24–26). However, waist-mounted accelerometers cannot assess actual postural changes and may misclassify SB and LPA (26). Additionally, although the reduction in SB in the intervention group partially supports the validity of the observed adherence, self-reported adherence to the short breaks may be affected by information bias (eg, recall and social desirability bias). Third, we included office workers from a single company in Japan. The participants were mainly women (92.4% in the intervention group) and were less sedentary during working hours than the office workers included in a previous systematic review (%SB at work: 62.9% versus 72.5%) (1). Additionally, the effectiveness of workplace health promotion may depend on organizational norms and climates, which vary across countries (12–14). Therefore, the generalizability of our results may be limited. Future studies should examine the effects of implementing short breaks in other populations, such as office workers in Western and developing countries and those who are more sedentary.

### Concluding remarks

A multicomponent workplace health promotion incorporating short active breaks from prolonged sitting may have long-term effectiveness in reducing occupational SB and increasing PA among office workers. However, the potential adverse effects on psychological distress and work engagement should be noted. While methodological limitations preclude definitively attributing the observed effects solely to the intervention, our findings suggest this program as a feasible approach to reducing SB and promoting PA during working hours among office workers. Further rigorous research is needed to assess its effectiveness and external validity before broad implementation can be endorsed.

### Protection of research participants

Consent for participation and the use of the collected data was obtained from all the study participants. The Ethical Committee of the Meiji Yasuda Life Foundation of Health and Welfare approved all the protocols (approval nos. 28006 and 30001).

## Supplementary material

Supplementary material
