# Supplementary material for: Effectiveness of short active breaks for reducing sedentary behavior and increasing physical activity among Japanese office workers: one-year quasi-experimental study
Source: Scand J Work Environ Health. 2025 Jun 26;51(4):312–22. doi: 10.5271/sjweh.4224 (PMC12282214; doi:10.5271/sjweh.4224)
Supplement: Supplementary material [file SJWEH-51-312-S001.pdf]

Effectiveness of short active breaks for reducing sedentary behavior and increasing physical activity among Japanese office workers: one-year quasi-experimental study[1]

by Naruki Kitano, PhD, Takashi Jindo, PhD, Kaori Yoshiba, MS, Daisuke Yamaguchi, MS, Yuya Fujii, PhD, Kyohsuke Wakaba, PhD, Kazushi Maruo, PhD, Yuko Kai, PhD,[2] Takashi Arao, PhD

1. Supplementary materials
2. Correspondence to: Yuko Kai, PhD, Physical Fitness Research Institute, Meiji Yasuda Life Foundation of Health and Welfare, 150 Tobuki, Hachioji, Tokyo 192-0001, Japan. [E-mail: y-kai@my-zaidan.or.jp]

Supplemental Table S1. The TREND Statement checklist

Paper Section/ Topic	Item No	Descriptor	Reported?	
			✓	Pg #
**Title and Abstract**				
Title and Abstract	1	• Information on how unit were allocated to interventions	✓	1
		• Structured abstract recommended	✓	1
		• Information on target population or study sample	✓	1
**Introduction**				
Background	2	• Scientific background and explanation of rationale	✓	2–3
		• Theories used in designing behavioral interventions	✓	2–3
**Methods**				
Participants	3	• Eligibility criteria for participants, including criteria at different levels inrecruitment/sampling plan (e.g., cities, clinics, subjects)	✓	4–5
		• Method of recruitment (e.g., referral, self-selection), including the sampling method if a systematic sampling plan was implemented	✓	4–5
		• Recruitment setting	✓	4–5
		• Settings and locations where the data were collected	✓	4–5
Interventions	4	• Details of the interventions intended for each study condition and howand when they were actually administered, specifically including:	✓	5–6
		○ Content: what was given?	✓	5–6
		○ Delivery method: how was the content given?	✓	5–6
		○ Unit of delivery: how were the subjects grouped during delivery?	✓	5–6
		○ Deliverer: who delivered the intervention?	✓	5–6
		○ Setting: where was the intervention delivered?	✓	5–6
		○ Exposure quantity and duration: how many sessions or episodes or events were intended to be delivered? How long were they intended to last?	✓	5–6, 9–10
		○ Time span: how long was it intended to take to deliver the intervention to each unit?	✓	5–6
		○ Activities to increase compliance or adherence (e.g., incentives)	✓	5–6
Objectives	5	• Specific objectives and hypotheses	✓	4
Outcomes	6	• Clearly defined primary and secondary outcome measures	✓	6–7
		• Methods used to collect data and any methods used to enhance thequality of measurements	✓	6–8
		• Information on validated instruments such as psychometric and biometricproperties	✓	6–8, Supplemental Table 3

Sample Size	7	• How sample size was determined and, when applicable, explanation of anyinterim analyses and stopping rules	✓	N/A
Assignment Method	8	• Unit of assignment (the unit being assigned to study condition, e.g.,individual, group, community)	✓	4
		• Method used to assign units to study conditions, including details of anyrestriction (e.g., blocking, stratification, minimization)	✓	5–6
		• Inclusion of aspects employed to help minimize potential bias induced dueto non-randomization (e.g., matching)	✓	8–9
Blinding (masking)	9	• Whether or not participants, those administering the interventions, and those assessing the outcomes were blinded to study condition assignment;if so, statement regarding how the blinding was accomplished and how it was assessed.	✓	N/A
Unit of Analysis	10	• Description of the smallest unit that is being analyzed to assess intervention effects (e.g., individual, group, or community)	✓	8–9
		• If the unit of analysis differs from the unit of assignment, the analytical method used to account for this (e.g., adjusting the standard error estimates by the design effect or using multilevel analysis)	✓	N/A
Statistical Methods	11	• Statistical methods used to compare study groups for primary methods outcome(s), including complex methods of correlated data	✓	8–9
		• Statistical methods used for additional analyses, such as a subgroup analyses and adjusted analysis	✓	8–9
		• Methods for imputing missing data, if used	✓	8–9
		• Statistical software or programs used	✓	9
**Results**				
Participant flow	12	• Flow of participants through each stage of the study: enrollment, assignment, allocation, and intervention exposure, follow-up, analysis (a diagram is strongly recommended)	✓	4–5, 9, Figure 1
		• Enrollment: the numbers of participants screened for eligibility,found to be eligible or not eligible, declined to be enrolled, and enrolled in the study	✓	9, Figure 1
		• Assignment: the numbers of participants assigned to a study condition	✓	4, 9, Figure 1
		• Allocation and intervention exposure: the number of participants assigned to each study condition and the number of participants who received each intervention	✓	4, 9, Figure 1
		• Follow-up: the number of participants who completed the follow-up or did not complete the follow-up (i.e., lost to follow-up), by study condition	✓	4, 9, Figure 1
		• Analysis: the number of participants included in or excluded from the main analysis, by study condition	✓	4, Figure 1
		• Description of protocol deviations from study as planned, along with reasons	✓	N/A
Recruitment	13	• Dates defining the periods of recruitment and follow-up	✓	5–6, Figure 1
Baseline Data	14	• Baseline demographic and clinical characteristics of participants in each study condition	✓	9, Table 1, Supplemental Figure 3
		• Baseline characteristics for each study condition relevant to specific disease prevention research	✓	N/A
		• Baseline comparisons of those lost to follow-up and those retained, overalland by study condition	✓	N/A
		• Comparison between study population at baseline and target population of interest	✓	N/A
Baseline equivalence	15	• Data on study group equivalence at baseline and statistical methods used to control for baseline differences	✓	7–8

Numbers analyzed	16	• Number of participants (denominator) included in each analysis for each study condition, particularly when the denominators change for different outcomes; statement of the results in absolute numbers when feasible	✓	9, Table 1
		• Indication of whether the analysis strategy was "intention to treat" or, if not, description of how non-compliers were treated in the analyses	✓	8–9
Outcomes and estimation	17	• For each primary and secondary outcome, a summary of results for each estimation study condition, and the estimated effect size and a confidenceinterval to indicate the precision	✓	9–10, Table 2–3
		• Inclusion of null and negative findings	✓	9–10, Table 2–3
		• Inclusion of results from testing pre-specified causal pathways through which the intervention was intended to operate, if any	✓	N/A
Ancillary analyses	18	• Summary of other analyses performed, including subgroup or restricted analyses, indicating which are pre-specified or exploratory	✓	9–10, Supplemental Table 4–5
Adverse events	19	• Summary of all important adverse events or unintended effects in each study condition (including summary measures, effect size estimates, and confidence intervals)	✓	9–10, Table 2–3
**DISCUSSION**				
Interpretation	20	• Interpretation of the results, taking into account study hypotheses, sources of potential bias, imprecision of measures, multiplicative analyses, and other limitations or weaknesses of the study	✓	10–13
		• Discussion of results taking into account the mechanism by which the intervention was intended to work (causal pathways) or alternative mechanisms or explanations	✓	10–13
		• Discussion of the success of and barriers to implementing the intervention,fidelity of implementation	✓	10–13
		• Discussion of research, programmatic, or policy implications	✓	10–13
Generalizability	21	• Generalizability (external validity) of the trial findings, taking into accountthe study population, the characteristics of the intervention, length of follow-up, incentives, compliance rates, specific sites/settings involved in the study, and other contextual issues	✓	13–14
Overall Evidence	22	• General interpretation of the results in the context of current evidence and current theory	✓	10–13

From: Des Jarlais, D. C., Lyles, C., Crepaz, N., and Trend Group (2004). Improving the reporting quality of non-randomized evaluations of behavioral and public health interventions: The TREND statement. *American Journal ofPublic Health* 94, 361-366. For more information, visit http://www.cdc.gov/trendstatement/

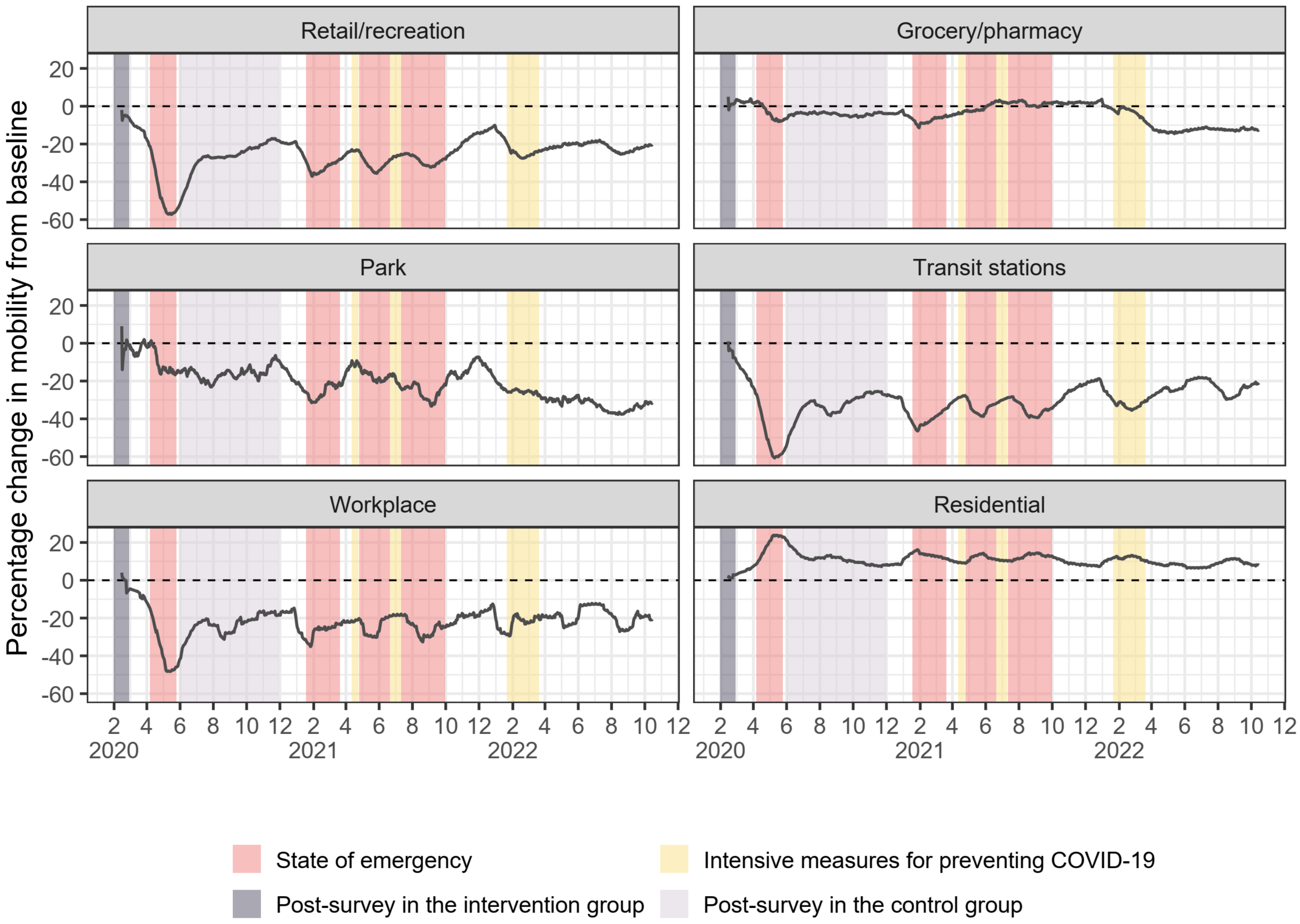

Supplemental Figure S1. Changes in mobility from the COVID-19 outbreak to the participants' enrollment periods in Tokyo, Japan. This figure was drawn using data from the Google Community Mobility Reports (Google, 2021; Gibbs et al., 2023). Mobility data were collected from Google Maps users who opted into sharing their location history with Google between February 15, 2020, and October 15, 2022 (data update was closed after this date). Mobility in each setting (e.g., workplace) is reported as the relative change from baseline calculated in the 4 weeks before the release of the dataset, from January 3 to February 6, 2020. Note: the Workplace graph represents the volume of mobility around the workplace rather than physical activity within the workplace. The percentage change in mobility is indicated as 30-day rolling average. The state of emergency and intensive infection control measures against COVID-19 included the following: 1) requests for residents to refrain from unnecessary and non-urgent outings, 2) school closures, 3) orders or requests to employers (e.g., restaurants and sports facilities) to close or shorten operating hours, and 4) restrictions on events with large numbers of people.

References:

- Google [Internet]. Overview - Community Mobility Reports Help. 2021 [cited 2024 Feb 26]. Available from: https://support.google.com/covid19-mobility/answer/9824897?hl=en
- Gibbs H, Ballantyne P, Cheshire J, Singleton A, Green MA. Harnessing mobility data to capture changing work from home behaviours between censuses. Geographical J. 2023;190:e12555.

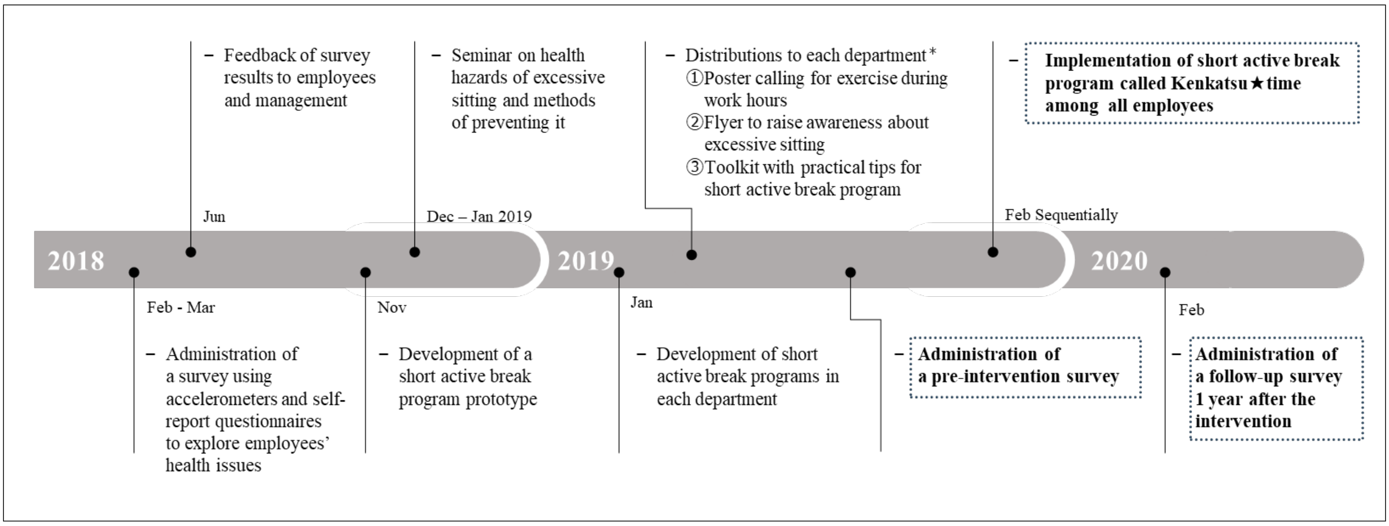

*Details are available in the Supplemental Material.

Supplemental Figure S2. Timeline of this study

A)

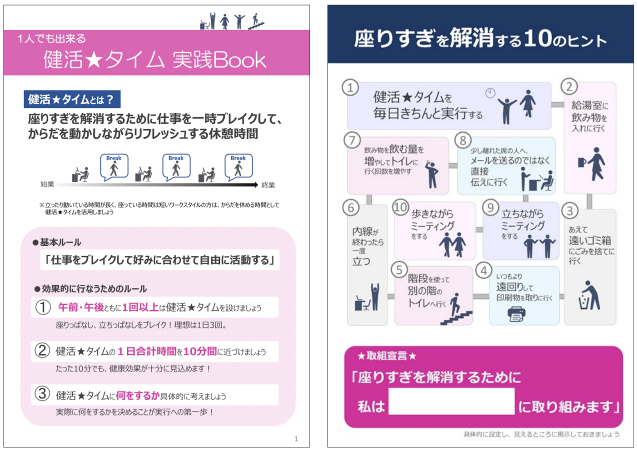

B)

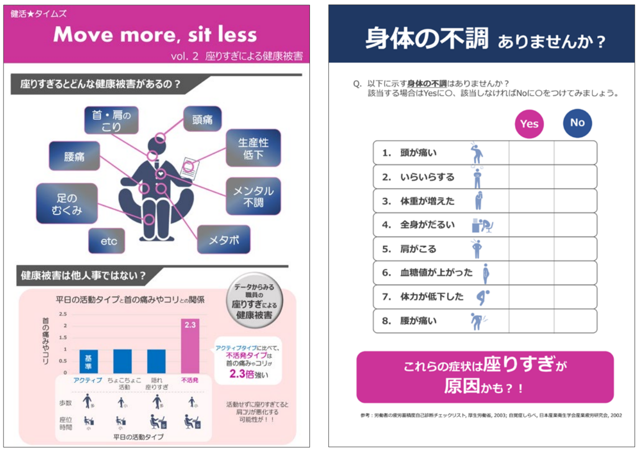

C)

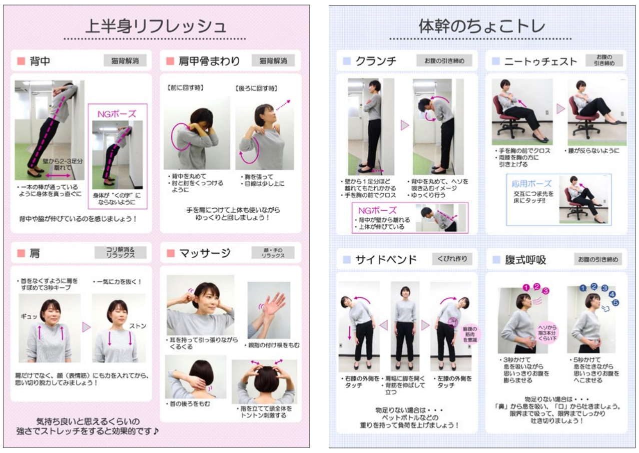

Supplemental Material S1. The educational material distributed to each department to facilitate adherence to the short-break program. A) Toolkit with tips for practicing Kenkatsu★Time. B) Flyer raising awareness regarding excessive sitting. C) Poster promoting stretching and muscle strength training during working hours and short active breaks

Supplemental Table S2. Detailed information on the short active break program in one department

Timing	Number of times	When	With what signal	Where	With whom	What
Baseline (February 2019)	1st	10:00−11:00 a.m.<br>5 minutes	Signal for “Kenkatsu★Time”		By group	Stretching, light walking, etc. for employees who have difficulty exercising while standing. Stretch exercises that can be performed while sitting are conducted
	2nd	14:00 hours<br>5 minutes	Signal for “Kenkatsu★Time”		Everyone	Exercise
	3rd	none				
Approximately 6 months after baseline (October 2019)	1st	10:00 hours<br>5 minutes	Music of radio exercise (using speaker in the office)	Around the seat	Everyone	Radio exercise
	2nd	14:00 hours<br>5 minutes	Exercise video (using large screen and speaker in the office)	Around the seat	Individually	Exercise while watching a DVD
	3rd	10:00 hours<br>(Every Wednesday)<br>5 minutes	When the person in charge calls out after the chime	Around the seat	By team	Stretching<br>(Predetermined original program)
Approximately 1 year after baseline (March 2020)	1st	10:00 hours<br>5 minutes	Music of radio exercise (using speaker in the office)	Around the seat	Everyone	Radio exercise
	2nd	14:00 hours<br>5 minutes	When the person in charge calls out after the chime	Around the seat	By team	Stretching<br>(Predetermined original program)
	3rd	none				

Supplemental Table S3. Detailed information regarding data collection for the secondary outcomes

Variables	Questions and response options
Psychological distress	Psychological distress was evaluated using the Japanese version of the K6 scale (Kessler et al., 2002; Furukawa et al., 2008). This scale comprises six psychological distress items, such as nervousness or restless, with occurrences in the previous month. The response options range from 0 to 4 (none of the time to all of the time), and the total score ranges from 0 to 24. The scale has high accuracy for diagnosing mood and anxiety disorders (area under the curve = 0.94) (Furukawa et al., 2008).<br>➢ Kessler RC, Andrews G, Colpe LJ, Hiripi E, Mroczek DK, Normand SLT, et al. Short screening scales to monitor population prevalences and trends in non-specific psychological distress. Psychol Med. 2002;32:959-76.<br>➢ Furukawa TA, Kawakami N, Saitoh M, Ono Y, Nakane Y, Nakamura Y, et al. The performance of the Japanese version of the K6 and K10 in the World Mental Health Survey Japan. Int J Methods Psychiatr Res. 2008;17:152-8.
Work engagement	Vigor of work engagement was assessed using the shortened Japanese version of the Utrecht Work Engagement Scale (UWES-9) (Schaufeli et al., 2006; Shimazu et al., 2008). The scale consists of three subscales with nine items that assess the current positive mental state for work, such as vigor, dedication, and absorption. This scale has high internal reliability (α = 0.91–0.92) and good test–retest reliability (ICC = 0.66) (Shimazu et al., 2008). Here, we used a vigor subscale of three items. Previous research confirms that these three subscales are strongly correlated with one another (r = 0.75–0.98) (Schaufeli et al., 2006). Therefore, the vigor subscale should reflect the overall work engagement.<br>➢ Schaufeli WB, Bakker AB, Salanova M. The measurement of work engagement with a short questionnaire: a cross-national study. Educ Psychol Meas. 2006;66:701-16.<br>➢ Shimazu A, Schaufeli WB, Kosugi S, Suzuki A, Nashiwa H, Kato A, et al. Work engagement in Japan: validation of the Japanese version of the Utrecht Work Engagement Scale. Appl Psychol. 2008;57:510-23.
Subjective job performance	Employees' subjective job performance was assessed using the following questions on absolute presenteeism from the World Health Organization Health and Work Performance Questionnaire (Kessler et al., 2003): "How would you rate your overall job performance on the days you worked during the past 4 weeks?" Response options range from 0 (worst possible work performance) to 10 (top work performance).<br>➢ Kessler RC, Barber C, Beck A, Berglund P, Cleary PD, McKenas D, et al. The World Health Organization Health and Work Performance Questionnaire (HPQ). J Occup Environ Med. 2003;45:156-74.

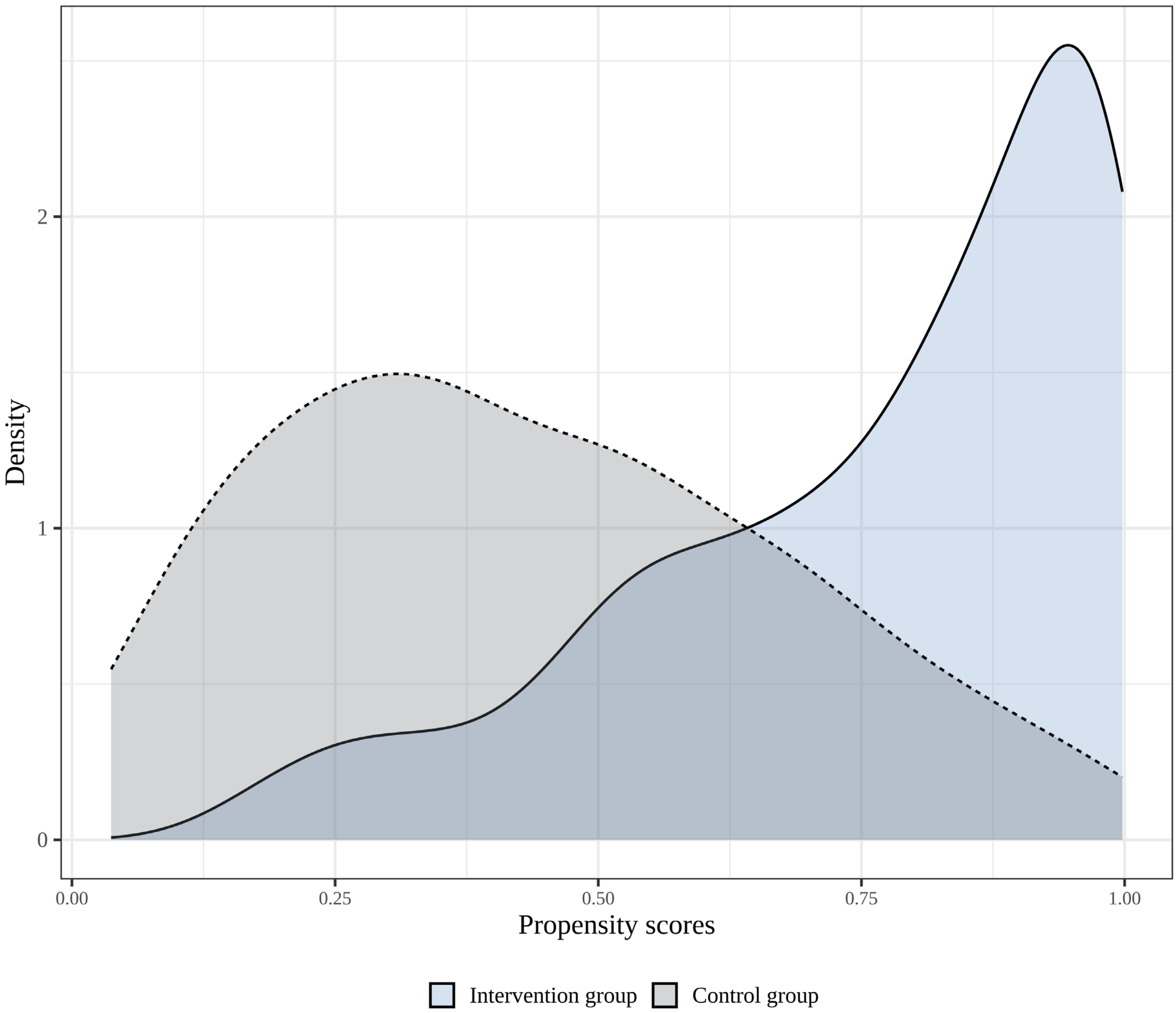

Supplemental Figure S3. Propensity score distribution in each group. Propensity scores were calculated using a logistic regression model with the group (intervention/control) as the dependent variable and age, sex, body mass index, job position, and baseline values for each outcome as independent variables.

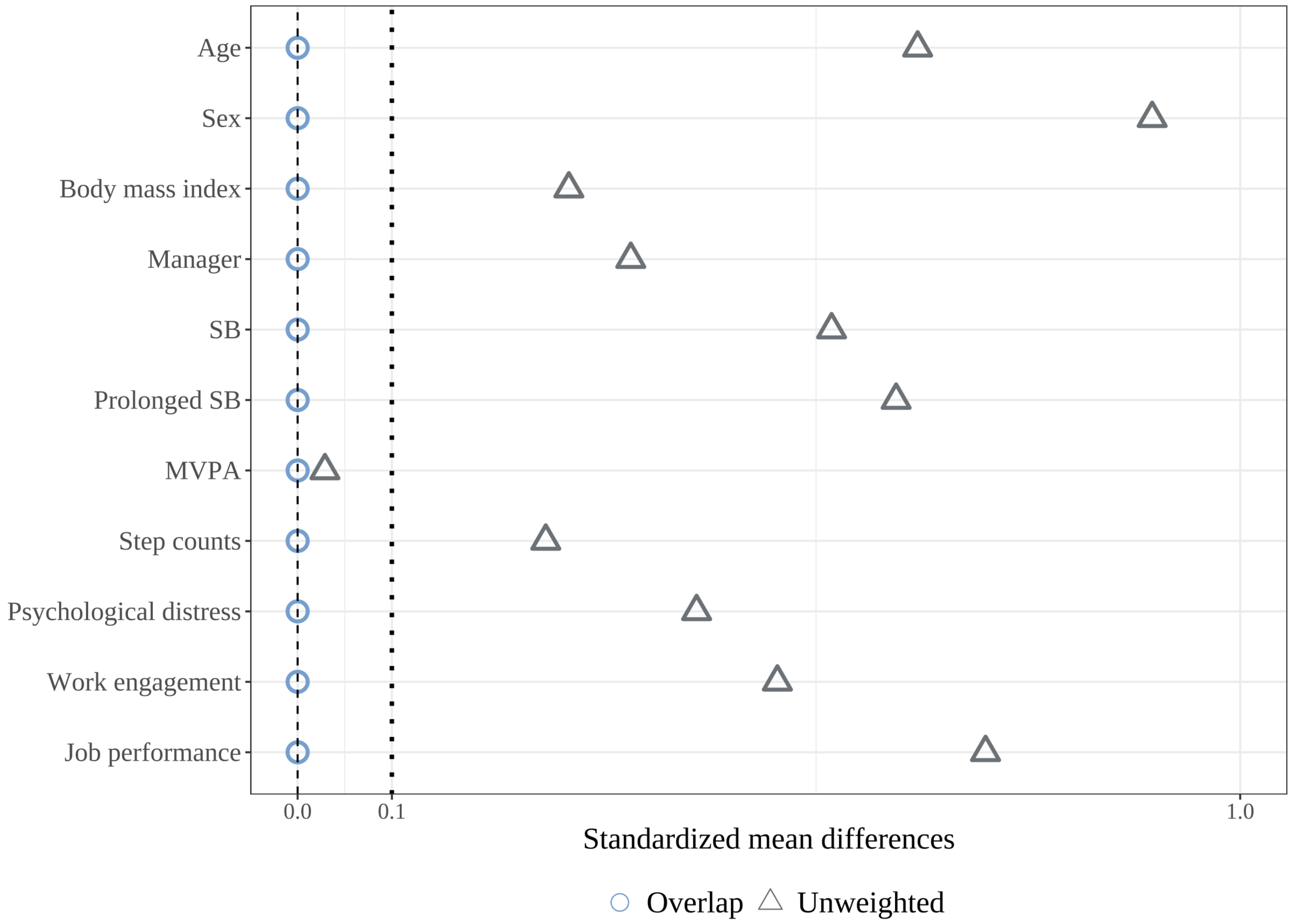

Supplemental Figure S4. Comparison of participant characteristics between the intervention and control group before and after propensity score weighting. SB, sedentary behavior; MVPA, moderate-to-vigorous–intensity physical activity. The movement behavior variables that were not listed on the y-axis were excluded from the propensity score model to avoid strong collinearity (e.g., light-intensity physical activity and frequencies of prolonged SBs).

Supplemental Table S4. Effect of short active breaks on primary outcomes in the complete case (intervention group, N = 114; control group, N = 290)

Outcome	Group	Pre (mean [SD])	Post (mean [SD])	Post - Pre difference (Estimate [95% CI])[a]	Group difference (Estimate [95% CI])	P for group difference
SB (min/8 hrs)	Intervention	308.1 (62.7)	288.2 (62.8)	-21.0 (-29.7—13.7)	-22.5 (-32.5—14)	0.001
	Control	341.4 (56.7)	342.8 (53.3)	1.5 (-3.1–6.2)		
Prolonged SB[b] (min/8 hrs)	Intervention	64.2 (67.7)	62.6 (57.2)	-1.0 (-11.3–9.8)	-6.4 (-18.5–6.5)	0.890
	Control	115.3 (83)	118.8 (80.9)	5.4 (-0.7–11.3)		
Prolonged SB[b] (counts/8 hrs)	Intervention	1.5 (1.4)	1.5 (1.2)	0.0 (-0.2–0.2)	-0.1 (-0.4–0.1)	0.909
	Control	2.4 (1.5)	26 (1.6)	0.1 (0.0–0.3)		
LPA (min/8 hrs)	Intervention	149.8 (56.7)	158.9 (55)	10.5 (2.8–19.3)	12.5 (3.6–22.8)	0.009
	Control	116 (53)	114.6 (50.6)	-2.0 (-6.5–2.3)		
MVPA (min/8 hrs)	Intervention	23.1 (13.1)	34 (16.2)	10.6 (8.0–12.9)	10.1 (7.1–12.8)	0.001
	Control	23.7 (13.5)	23.7 (12.7)	0.5 (-0.9–1.9)		
Bouted PA[c] (min/8 hrs)	Intervention	75.8 (34.2)	81.7 (33.5)	6.8 (1.7–11.4)	8.9 (2.9–14.5)	0.008
	Control	57.6 (33.)	57.1 (30.5)	-2.0 (-5.1–0.8)		
Bouted PA[c] (counts/8 hrs)	Intervention	17.1 (7.0)	18.1 (6.6)	1.0 (0.0–2.0)	1.7 (0.4–2.8)	0.046
	Control	13.2 (6.9)	13.0 (6.3)	-0.6 (-1.2–0.0)		
Step count (steps/8 hrs)	Intervention	3834.5 (1313.6)	4988.8 (1708.6)	1132.9 (852.4–1410.7)	1134.7 (809.6–1452.5)	0.001
	Control	3597.2 (1385.8)	3648.3 (1381.3)	-1.8 (-172.7–173.7)		

SB, sedentary behavior, LPA, light-intensity physical activity, MVPA, moderate-to-vigorous-intensity physical activity; SD, standard deviation; CI, confidence interval. [a]Because these values are adjusted for covariates, they do not exactly match the difference between the values in the Pre and Post columns, which are arithmetic means. [b]Unbroken sitting bouts ≥30 minutes. [c]Continued physical activity ranging from 3 to 10 minutes.

Supplemental Table S5. Effect of short active breaks on secondary outcomes in the complete case (intervention group, N = 114; control group, N = 290)

Outcome [a]	Group	Pre (mean [SD])	Post (mean [SD])	Post - Pre difference (Estimate [95% CI])[b]	Group difference (Estimate [95% CI])	P for group difference
Psychological distress (points)	Intervention	5.1 (5.2)	6.9 (5.7)	1.8 (1.1–2.6)	1.6 (0.7–2.6)	0.001
	Control	3.1 (4)	3.5 (4.2)	0.2 (-0.4–0.8)		
Work engagement (points)	Intervention	2.2 (0.9)	2.1 (1)	-0.1 (-0.3–-0.0)	-0.2 (-0.4–0)	0.046
	Control	2.9 (1.5)	2.9 (1.5)	0.1 (-0.0–0.3)		
Job performance (points)	Intervention	5 (1.3)	5.3 (1.5)	0.1 (-0.2–0.5)	-0.2 (-0.6–0.2)	0.404
	Control	6.1 (1.6)	6.2 (1.5)	0.4 (0.1–0.6)		

SD, standard deviation; CI, confidence interval. [a]Larger values indicate higher levels of psychological distress, work engagement, and job performance. [b]Because these values are adjusted for covariates, they do not exactly match the difference between the values in the Pre and Post columns, which are arithmetic means.
